# Study on effect of collaborative governance participation willingness of online food delivery platform restaurants and consumers from perspective of control theory: Based on moderating effects of perceived risks

**DOI:** 10.3389/fpsyg.2023.1149538

**Published:** 2023-03-14

**Authors:** Xiaoting Dai, Ke Qin, Linhai Wu

**Affiliations:** ^1^School of Business, Jiangnan University, Wuxi, China; ^2^Institute for Food Safety Risk Management, Jiangnan University, Wuxi, China

**Keywords:** online food delivery, platform governance, food safety, control theory, perceived risk

## Abstract

The popularization of the Internet and the convenience of e-commerce are driving the online restaurant industry’s rapid development of worldwide. However, serious information asymmetries in online food delivery (OFD) transactions not only aggravate food safety risks, resulting in simultaneous government and market failures, but also intensify consumers’ perceived risks. This paper innovatively constructs a research framework for the governance participation willingness of OFD platform restaurants and consumers under the moderating effects of perceived risks from the perspective of control theory and then develops scales for analyzing the governance willingness of both restaurants and consumers. Using data collected through a survey, this paper explores the effect of control elements on governance participation by restaurants and consumers and analyzes the moderating effects of perceived food safety risks. Results showed that both government regulations and restaurant reputation (formal control elements) and online complaints and restaurant management response (informal control elements) can increase governance participation willingness among both platform restaurants and consumers. The moderating effects of perceived risks are partially significant. When the risks perceived by restaurants and consumers are strong, government regulation and online complaints can more effectively boost the governance participation willingness of restaurants and consumers, respectively. At this moment, consumers’ willingness to pursue problem solving through online complaints is evidently enhanced. Accordingly, the perceived risks and the online complaints jointly motivate restaurants and consumers to participate in governance activities.

## 1. Introduction

In recent years, the platform economy has risen rapidly worldwide, promoting the quick development of the online restaurant industry – a novel food consumption format (Kassi and Lehdonvirta, 2018; Cho et al., 2019; Cheng et al., 2021). Different types of online food delivery (OFD) platforms, represented by Uber Eats in the US, Deliveroo in UK, Swiggy in India, iFood in Brazil, and Eleme and Meituan in China, have been growing rapidly (Gunden et al., 2020; Li et al., 2020). OFD has gradually become an important method of daily food purchases; in particular, its popularity boomed as part of prevention and control of the COVID-19 pandemic (Gao et al., 2020; Oncini et al., 2020; Li et al., 2022; Lu et al., 2022). The online restaurant industry has relatively optimistic development prospects for the future. According to estimations, the global OFD market will continue growing at a mean annual rate of 8.28% during 2022–2026. China’s current OFD consumption has reached a huge scale of 544 million person-times (China Internet Network Information Center (CNNIC), 2022), and over 40% of restaurants now provide consumers with parallel online and offline services (Huaan Securities, 2021).

The quickly developing, large-scale, multi-user OFD platforms serve to directly connect numerous dispersed food producers and operators with millions upon millions of consumers. However, because OFD transactions are characterized by virtuality, invisibility, and complexity, OFD platforms can increase information asymmetries (Du et al., 2019), bringing new concerns to governments’ traditional food regulatory system and new challenges to the traditional reputation-based market governance mechanism. Simultaneous government and market failures may occur in OFD food safety risk governance (Zhang, 2021), which infringes consumers’ right to health. Accordingly, it has been of great urgency to establish and improve a collaborative governance mechanism for OFD food safety risk participated in by all stakeholders including restaurants and consumers.

China has the largest OFD market, whose scale is unmatched by those in other countries (Statista, 2023). In this sense, studying the food safety risk governance of China’s OFD platforms is somewhat unique, forward-looking, and representative. The contribution of this paper lies in innovatively constructing a research framework for measuring the governance participation willingness by OFD platform restaurants and consumers under the moderating effects of perceived risks from the perspective of control theory. This study also contributes to the literature by independently developing scales for analyzing the governance participation willingness of restaurants and consumers based on the Chinese context, verifies the scales’ effectiveness, and studies the influences of control elements and perceived risk elements on both platform restaurants’ and consumers’ willingness to participate in governance by analyzing collected sample data using the structural equation model (SEM) and latent moderated structure (LMS) model in a combined manner.

## 2. Literature review

Given OFD platforms’ ability to cater to consumers’ diversified and convenient food consumption demands, they have spurred the emergence of large numbers of ghost kitchens^1^ existing in the UK, US, India, and China with the rapid development of the online restaurant industry (Nita, 2019; Belleri, 2020). These ghost kitchens pose food safety risks, and their potential to cause health issues among consumers is a concern (Li et al., 2020). Links such as packaging and distribution are included in OFD, unlike traditional food consumption formats, so there is a risk of secondary food contamination. For example, food overflow to the packaging box often occurs during distribution of restaurant food. In particular, residues in the distribution box can become a culture medium for microbial growth under hot weather conditions; a distributor whose sanitary conditions may not meet standards has many opportunities to make contact with and contaminate food. In addition, it is possible to contaminate food *via* the packaging materials in the added packaging link, and so on (Zhang, 2021). More seriously, there are many moral hazards for greater economic benefits in the online food transaction market; for example, bad restaurants can generate false data by dint of the network’s virtuality, which can universally increase food safety risks (Wei and Yao, 2020).

Food is a credence product. Even after buying and eating an item of food, consumers may not be able to identify relevant safety information (McCluskey, 2000; Grunert, 2002). There is an unavoidable information asymmetry between consumers and producers (Ortega et al., 2011). Different from traditional food transactions, OFD transactions are a novel economy form constructed based on Internet information technology and are thus characterized by virtuality (Martinez-Navarro et al., 2019), invisibility (Atif, 2002), and complexity (Alsaad et al., 2018), etc. The platforms and restaurants possess more food safety information, and they take a strategy of not disclosing information as far as possible based on their own interests, which aggravates the information asymmetry among the stakeholders (Langley and Leyshon, 2017). This provides restaurants with more opportunities to take opportunistic behaviors^2^ to seek high earnings (Rees, 2020). Although the government has applied some regulatory methods used for offline food to the management of OFD platforms (for example, restaurants are required to provide store pictures and a business license, so as to alleviate some information asymmetry), the effect is relatively limited given the virtual environment, and the difficult problem of information asymmetry is hard to solve (Ding et al., 2022). Further, because the network directly connects the producers, operators, and consumers, online restaurant food can appear on the dining-tables of thousands of households at lower costs than offline food. It then avoids the traditional government regulation system, aggravating government regulatory failure (Wu et al., 2019; Xiao and Yang, 2022).

Information asymmetries further aggravate market failures in the online restaurant industry. For example, asymmetries may suppress the function of the reputation mechanism. Because food has the characteristics of experience goods, consumers can only understand it only after buying and eating it (Grunert, 2005). Nevertheless, because food is an inelastically demanded living consumable and is characterized by long-term and repeated buying behavior, consumers can obtain information and identify food quality through a reputation mechanism. A reputation mechanism is essentially a market signal searching and screening mechanism, and it can affect consumers’ cognition of food quality to give rise to a governance function of “voting with your feet” (Pichler and Wilhelm, 2001; Benson et al., 2020). However, given that food is also a credence product, the platforms mastering and controlling massive amounts of data are not willing to disclose more information based on their own economic benefits. This leaves consumers disadvantaged and unable to make objective cognitions about restaurants on the platforms. Accordingly, the reputation mechanism’s governance function fails to work effectively (Segerson, 1999), and restaurants, with their information advantages, will continue to engage in moral hazard behaviors to obtain excess economic returns (Jin et al., 2016; Liu, 2018; Fan and Qiu, 2021).

The serious information asymmetry existing in OFD transactions not only intensifies simultaneous government and market failures but also enhances consumers perceived food safety risks (Filieri et al., 2018; Ahani et al., 2019). The concept of perceived risk is defined as the risk degree perceived by a consumer when making a decision to buy a product or service (Zhu et al., 2017). The risks perceived by consumers in online consumption behaviors originate from the guesswork involved arising from the complexity, virtuality, and uncertainty of online transactions (Tham et al., 2019). Further, compared with ordinary online goods transactions, OFD transactions are related to human health, so they not only enhance consumers’ awareness for perceived risks but also have a greater effect on consumers’ willingness to buy (Pappas, 2016; Munikrishnan et al., 2021; Pillai et al., 2022).

The emergence and development of the online restaurant industry only began 10 years ago (Zhao et al., 2021), but scholars have performed many active explorations regarding how to govern OFD food safety risks. He and Zhu (2020) pointed out that the risks in online consumption originate from inadequate government regulation, restaurants’ moral hazards, and consumers’ ineffective complaints against the background of information asymmetry. Yin et al. (2020) believed that the OFD regulation mode of combined government intervention and market mechanism based on information sharing can make up for simultaneous government and market failures. Wang et al. (2020) argued that platform self-discipline and government regulation strength affect restaurants’ strategy choices. Zhao (2018) believed that mandatory government regulation and societal involvement in supervision help ensure that platforms uphold their social responsibilities. Wood et al. (2017) and Wood (2018) thought that lack of either external or internal supervision may result in overexpansion of platforms and induce restaurants’ moral hazards. Thus, existing studies have consistently shown that OFD food safety risk governance needs the participation of multiple stakeholders. Barinda and Ayuningtyas (2022) found in study on food safety risk control coefficient that all of the sharing of regulatory resources, the balance among stakeholders and the continuous update of scientific knowledge are extremely complex. The OFD transactions achieve direct connection of “producer and operator – consumer” by Internet technology, so the food avoids the traditional government supervision system and directly reaches dining-tables, causing the traditional regulation method with long-standing problems to be difficult to work (Zhao, 2017; Wu et al., 2019).

However, existing studies were carried out mostly from a single perspective, i.e., government or platform, and did not consider the situation of more serious information asymmetries existing in the online restaurant industry, and they did not incorporate all stakeholders into one system to provide a complete picture from the perspective of joint governance participation based on respective responsibilities, forming a governance system. Further, few studies have incorporated into their research framework the perceived risk element that can significantly influence platform governance effects. Based on this, this paper attempts to fill up the gaps in the above references and the main efforts and contributions of this paper are as follows: Based on the background of rapid development, large scale, numerous users and high penetration of China’s online restaurant industry and the objective reality of simultaneous government and market failures in OFD platform governance, and oriented by how to alleviate information asymmetries in the online restaurant industry to reduce food safety risks, this paper incorporates all stakeholders into the regulation governance system to innovatively construct a research framework for the governance participation willingness of OFD platform restaurants and consumers under the moderating effects of perceived risks from the perspective of control theory. This study also independently develops scales for the governance participation willingness of restaurants and consumers and verifies these scales’ effectiveness. Using collected sample data, this paper analyzes the influences of control elements on governance participation willingness of the restaurants and consumers using SEM and analyzes the moderating effects of perceived risks on the governance participation willingness using the LMS model.

## 3. Research framework, model construction, and research hypotheses

### 3.1. Research framework

Control is defined as a mechanism through which a controller or a group of controllers of one type influence controllees in multiple ways so that the controllees constantly perform adjustment and optimization according to the controllers’ objectives (Ouchi, 1979). All social problems can be considered to occur in controlled social systems. Control theory is devoted to placing interdependent and mutually constrained social systems under control, and regulating the mutual relationships between controllers and controllees through such means as optimization, prevention, and control so that the systems operate according to the objectives (Eisenhardt, 1985; Kirsch, 1997). Control mechanisms mainly include formal control and informal control (Zheng et al., 2019). Scholars including Leoni and Parker (2018), Gol et al. (2019), and Pan et al. (2022) proposed a concept of a platform ecosystem^3^ and introduced control theory into platform ecosystem governance.

**Figure 1:**
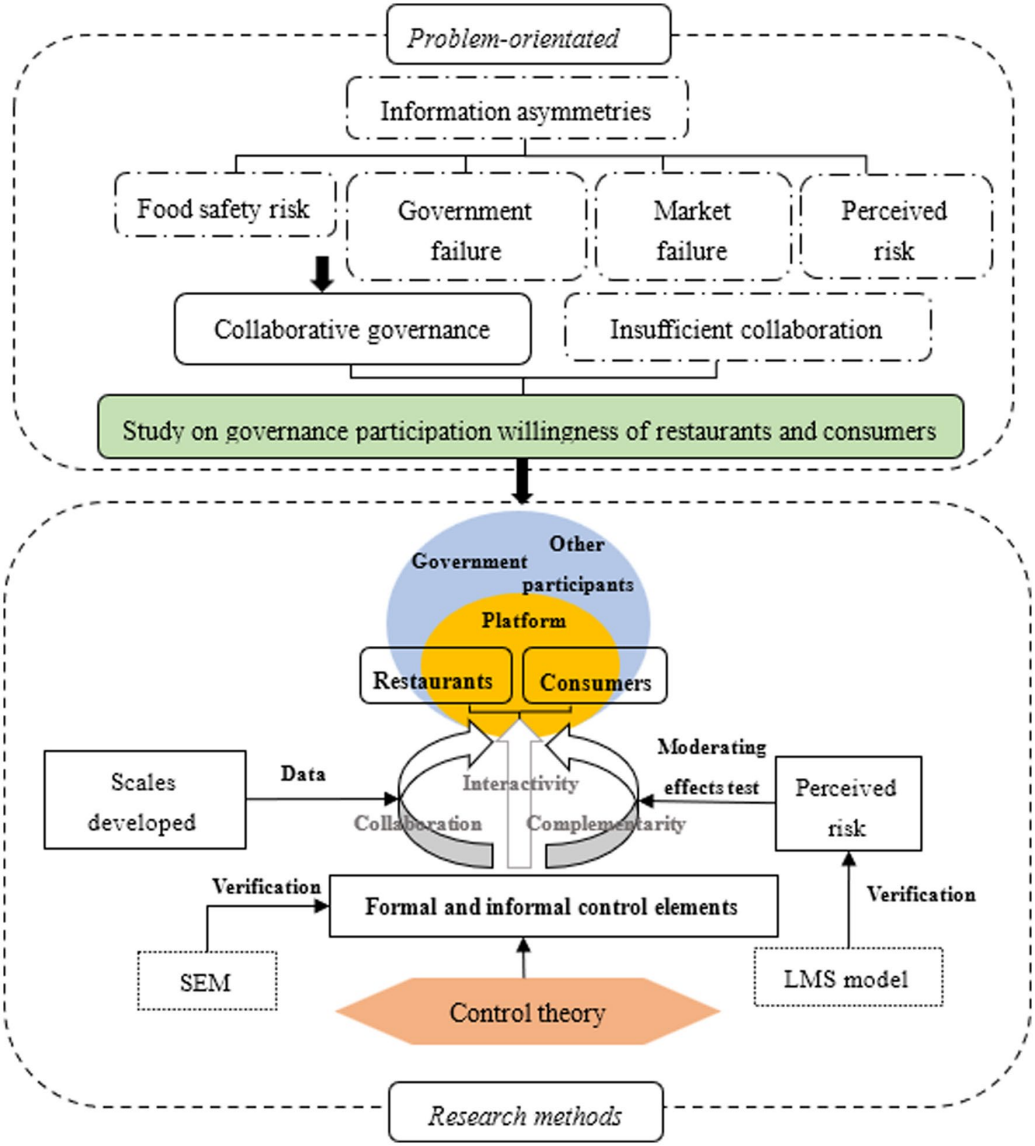
Research framework for governance participation willingness of OFD platform restaurants and consumers. The figure shows the primary cause analysis of the research. The framework, the theory, models and data employed of the research are also illustrated in the figure.

Referencing the above research conclusions, this study regards an OFD platform as an ecosystem, which not only is controlled by external stakeholders including the government and social organizations, but also responds to influences of internal stakeholders including OFD platform, restaurants, and consumers (Rietveld et al., 2019). Therefore, the question of how to coordinate internal and external platform stakeholders becomes a key element for OFD food safety risk governance (Tiwana et al., 2010). Jiang et al. (2021) divided the management mechanisms of a platform ecosystem into formal control mechanisms, including government regulation, and platform management, and informal control mechanisms involving restaurants and consumers. On the above basis, this paper incorporates all stakeholders into one system by combining formal and informal control and embeds different types of control elements exhibiting complementarity, collaboration, and interactivity into the platform governance mechanism through all platform stakeholders using control theory. The proposed approach aims to promote information communication and function coordination among all stakeholders, thereby achieving the governance objective of alleviating information asymmetries to reduce risks. Consequently, constructing an ecological platform based on the control theory provides a new perspective for OFD food safety risk governance. Meanwhile, referencing the research conclusion that perceived risks can significantly influence ecosystem control results in information technology governance (ITG) (Liu and Deng, 2015) and the viewpoint that platform risk governance and perceived risks are inseparable, as proposed by Zanetta et al. (2021), as well as considering that perceived risks can significantly affect consumers’ willingness to buy (Cai and Leung, 2020), this paper incorporates perceived risks as a moderating variable into an OFD platform. Accordingly, this paper proposes the research framework shown in Figure 1.

### 3.2. Model construction

**Figure 2:**
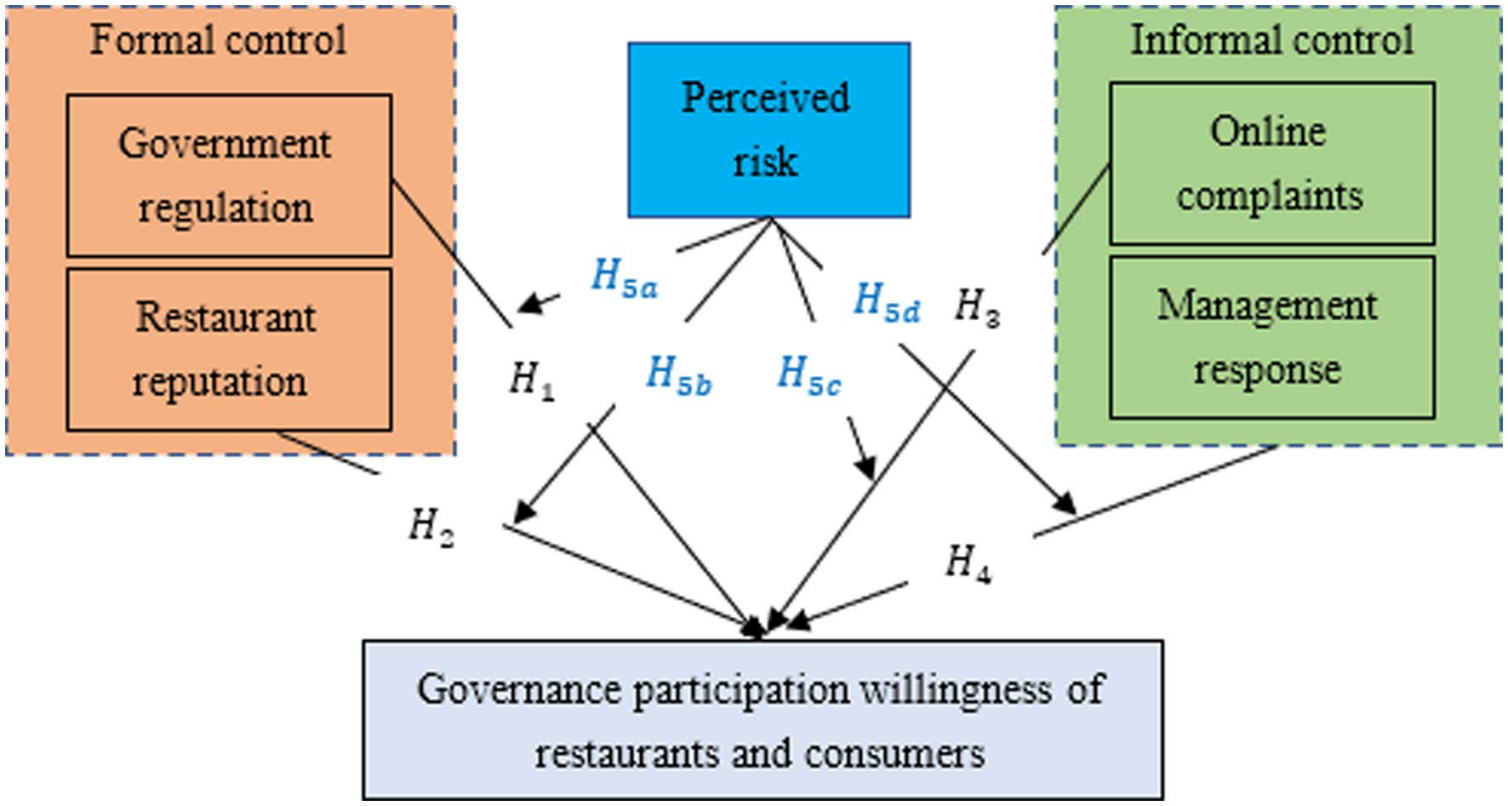
Research model for governance participation willingness of restaurants and consumers. The figure illustrates the influences of formal and informal control elements on governance participation willingness by restaurants and consumers, respectively. It also shows the moderating effects of perceived risks by restaurants and consumers on these influences.

Based on the research framework in Figure 1, the control elements are divided into formal and informal ones (Kirsch, 1996). The elements emphasizing reward or punishment, i.e., government regulation and restaurant reputation, are defined as formal control elements, and other elements that allow stakeholders to achieve participation through self-management, i.e., consumer online complaints and restaurant management responses, are defined as informal control elements. The research model shown in Figure 2 was constructed accordingly. Corresponding hypotheses are proposed based on the literature study and objective facts to test the influences of the control elements and perceived risks as moderating variables on governance participation willingness by restaurants and consumers.

### 3.3. Research hypotheses

#### 3.3.1. Influences of formal control elements on governance participation willingness of restaurants and consumers

Process and result controls are classified as formal control elements given their emphasis on reward or punishment as a means to control the system (Liu et al., 2017). The government is the supplier of systems, government regulations on OFD platform constitute the control mechanism in platform governance, and the control objectives are regarded as the platform governance criteria (Janowski et al., 2018). Through arrangement of systems, the government guides platforms to meet for the public value of ensuring food safety while achieving their own economic benefits (Martin et al., 2017; Ganapati and Reddick, 2018). Therefore, this paper takes government regulation as one process control element. Accordingly, the following hypothesis is proposed:

*H_1_*: Government regulation has a positive influence on the governance participation willingness of OFD platform restaurants and consumers.

Reputation, as society’s cognition about an enterprise’s past behaviors (Kreps and Wilson, 1982), affects that enterprise’s survival and development (Rayner, 2004; Brown et al., 2006). Therefore, reputation can be taken as a result control element to measure the control effect (Jiang et al., 2021). In addition, reputation has an implicit incentive effect on consumers’ evaluations (Sparks et al., 2016). It is more important that reputation can constrain restaurants to reduce their opportunistic behaviors and drive them to make efforts to improve goods and service quality so as to gain consumers’ trust (Sthapit, 2019). Restaurants’ pursuit for reputation can serve as a mechanism by which they internalize interests (Toubia & Stephen, 2013), motivating OFD platform stakeholders to participate in governance. Accordingly, the following hypothesis is advanced:

*H_2_*: Restaurant reputation has a positive influence on the governance participation willingness of OFD platform restaurants and consumers.

#### 3.3.2. Influences of informal control elements on governance participation willingness of restaurants and consumers

Informal control elements are those elements that help achieve governance objectives through self-management, etc., with a focus on social or human elements’ influences on platform management systems (Choudhury and Sabherwal, 2003). As an element related to the people interacting with the platform ecological environment, consumer online complaints can be regarded as an informal control element (Jiang et al., 2021). The serious information asymmetries existing in online platforms make consumers increasingly judge product quality and suitability based on online evaluations from previous consumers (Kwark et al., 2014); accordingly, online complaints can influence the economic returns of restaurants or platform (Sthapit and Bjork, 2019) and also positively affect other consumers’ complaints (Dolan et al., 2019), driving the restaurants to take measures for improving the quality of its commodities and service (Sahin et al., 2017; Moon et al., 2019). Accordingly, the following hypothesis is proposed:

*H_3_*: Online complaints have a positive influence on the governance participation willingness of OFD platform restaurants and consumers.

A restaurant’s active response to consumers’ evaluations can enhance consumers perceived credibility of that restaurant, thereby affecting existing and potential consumers’ evaluations and weakening the negative reputation influence of negative evaluations on the restaurant (Chen et al., 2019; Armas-Cruz et al., 2022). Industrial management under modern information technology is gradually changing from a management mode of passively knowing consumers’ evaluations to one of actively responding consumers and interacting with them; this mode has proven to help restaurants to increase benefits, etc. (Xie et al., 2017). Accordingly, the following hypothesis is offered:

*H_4_*: Restaurant management response has a positive influence on the governance participation willingness of OFD platform restaurants and consumers.

#### 3.3.3. Moderating effects of risks perceived by restaurants and consumers

Government regulation resources are objectively scare, and the government, as a party disadvantaged in terms of possessing OFD production and operation information, has bounded rationality, which results in limited ability of the government for platform regulation (Antle, 1999; Moruzzo et al., 2020). Therefore, the government must adjust its regulation methods and strength according to the risk degree of the platform operation while also carrying out its regulation responsibility over OFD platforms. Accordingly, the following hypothesis is proposed:

*H_5a_*: The positive influence of government regulation on the governance participation willingness of OFD platform restaurants and consumers is moderated by perceived risks, and the effect is positively correlated with the perceived risks.

When consumers and restaurants, etc. perceive high risks for participating in an OFD platform, negative emotions will be caused to different degrees. For example, negative media reports or adverse evaluations, etc. will impact restaurant reputations (Syed, 2019). Therefore, when the increase of perceived risk leads to reputation damage, the governance participation willingness of restaurants may be raised. Accordingly, the following hypothesis is advanced:

*H_5b_*: The positive influence of restaurant reputation on the governance participation willingness of OFD platform restaurants and consumers is moderated by perceived risks, and the effect is positively correlated with the perceived risks.

An OFD platform directly connects dispersed food producers, operators, and consumers based on the Internet; in such circumstances, online complaints become one important method for solving food quality and service problems occurring in online consumption. When consumers’ perceived risks increase, the number of online complaints will rise, and the governance willingness of the restaurant and consumers will be enhanced. Accordingly, the following hypothesis is stated:

*H_5c_*: The positive influence of online complaints on the governance participation willingness of OFD platform restaurants and consumers is moderated by perceived risks, and the effect is positively correlated with the perceived risks.

The rapid rise of OFD platforms is based on the quick development of information technology. Therefore, restaurants can reply rapidly to the online complaints or various questions from consumers during the platform operation. The stronger the risks perceived by a restaurant, the more attention the restaurant may pay to solving consumers’ online complaints, making improvement to guard against consumers’ adverse evaluations, and so on, to reduce current losses and achieve long-term benefit objectives as far as possible. Accordingly, the following hypothesis is proposed:

*H_5d_*: The positive influence of restaurant management responses on the governance participation willingness of OFD platform restaurants and consumers is moderated by perceived risks, and the effect is positively correlated with the perceived risks.

## 4. Research method and data collection

### 4.1. Development of scales for governance participation willingness of restaurants and consumers

An OFD platform is a typical bilateral market^4^, and restaurants and consumers are the most direct stakeholders. Accordingly, this paper develops two scales for governance participation willingness, in the online Supplementary Tables S1, S2, for these two research objects, respectively. These scales have roughly similar contents, with only a few entries being different. Each scale has six dimensions, i.e., government regulation, restaurant reputation, online complaints, management response, perceived risk, and governance participation willingness, which are measured using a seven-point Likert scale. The items in the government regulation dimension were designed mainly by referencing the research results obtained by Lewis et al. (2003) and Xu et al. (2012). The items in the restaurant reputation, online complaint, management response, perceived risk, and governance participation willingness dimensions were based on the research results obtained by McKnight et al. (2002), Wu (2013), Pavlou and Gefen (2004), Leung et al. (2019), and Jiang et al. (2021), respectively. Each dimension contains three items, and each scale contains 18 items in total.

As described in detail in the next sections, we collected data through a questionnaire survey. First, to analyze the scale items and screen substandard items so as to verify the reliability and validity of the scales, we then conducted a pre-survey and collected 190 valid questionnaires in total. The formal questionnaire survey then was carried out on the basis that the pre-survey questionnaire was determined to have good reliability and validity, and 946 valid questionnaire samples were collected in total. The study in this paper is performed gradually according to the following steps: questionnaire design, pre-survey and data collection, verification of scale rationality, formal survey and data collection, and verification of collection reliability.

To ensure the scientific validity and reliability of the survey, it was performed by Wuxi Market Supervision Bureau, China. Wuxi Market Supervision Bureau randomly distributed questionnaires to restaurants with an established presence on the OFD platform, and the questionnaires were filled in by legal persons representing the restaurants or their designated persons. The consumer survey was carried out by creating an online link to the survey website, and the link was shared on social media to be propagated to more consumers. The respondents selected in this paper had to have the experience in purchasing and consuming OFD food and those not meeting this requirement were rejected for the survey. Before respondents could start to fill in the survey questionnaires, they provided informed consent for the survey by reading the first page of the survey questionnaire.

### 4.2. Questionnaire pre-survey and scale verification

#### 4.2.1. Analysis of scale items

Before the formal survey in this paper, pre-survey and data collection were performed and scale reliability was verified, for the purpose of preparing for the formal survey, to verify whether the designed scale is rational. Compared with formal survey, the pre-survey had a small sample size. The sample size for the pre-survey in this paper was 190, including 81, and 109 samples from restaurant legal persons (operators), and consumers, respectively. The analysis of scale items was as follows: items were ranked by total score, and a high-score and a low-score group were eliminated by screening. Then, we conducted mean score difference significance testing between the item scores in the high-score group and those in the low-score group using an independent samples *t*-test. If the critical ratio (CR) of an item is less than 3 and *p* > 0.05, this item should be deleted; otherwise, it should be retained (Wu, 2000). As can be discerned in the online Supplementary Table S3, the CR values of all items in the scales of participation willingness designed in this study are more than 3, with the significance level reached (*p* < 0.001), indicating that all measurement items in the developed scales are feasible.

#### 4.2.2. Reliability test

The corrected item total correlation (CITC)^5^ values and Cronbach’s *α* values^6^ of the scale items were calculated with the software SPSS to verify the reliability of the scales. If the CITC value is less than 0.4 or α value is less than 0.7, the item should be deleted; otherwise, it should be retained (Wu, 2000). As can be discerned in the online Supplementary Table S4, the CITC values of all scale items in the pre-survey samples are more than 0.4, deletion of any item cannot increase the α values of subscales, and all α values conform to the criterion of more than 7, so all measurement items in the scales for governance participation willingness of restaurants and consumers are suitable.

#### 4.2.3. Exploratory factor analysis

A measure test was conducted on pre-survey samples using a Kaiser-Meyer-Olkin (KMO)^7^ test. This aimed to determine whether the designed scale items satisfy the conditions for factor analysis. The results of pre-survey samples of restaurants and consumers show that the KMO values of all six dimensions are more than 0.7, indicating that there are many common factors between variables and the factor analysis is suitable. A mature scale where the scale facet structure has been determined and factor analysis of individual dimension can be conducted for subscale items, respectively (Wu, 2000), was used in the questionnaire formulation process in this study. Accordingly, in the pre-survey, exploratory factor analysis was conducted for six dimensions using the principal component analysis method coupled with the maximum variance orthogonal rotation method to determine the factor structure of each potential variable. The three items of each six dimensions in the scale can be extracted as one principal component, and the cumulative variance interpretation rates of the results of both pre-survey questionnaires are more than 60%. The factor loading coefficient^8^ values of all items conform to the criterion of more than 0.5, indicating that all items constituting the scale have significant contributions and clearly form six dimensions. Therefore, all items in the proposed scale for restaurant and consumer governance participation willingness are suitable.

### 4.3. Questionnaire survey and scale verification

#### 4.3.1. Reliability and validity test

The Cronbach’s *α* value and CITC were selected to conduct reliability test for the formal survey sample data. Based on Tables 1, 2, the *α* values of both questionnaire samples for restaurants and consumers are greater than 0.7, indicating that the sample data have good reliability and internal consistency. The CITC value of each item in the questionnaire samples for restaurants and consumers is more than 0.4, further indicating that the scales have good reliability.

**Table 1:** Scale properties for restaurants.

Dimension	Item	Cronbach’s α	CITC	Communality	Factor loading	Composite reliability	AVE
Government regulation	GR1	0.866	0.750	0.782	0.802	0.866	0.683	GR2		0.757	0.854	0.88			GR3		0.729	0.768	0.796		
Restaurant reputation	RR1	0.861	0.774	0.776	0.898	0.862	0.676	RR2		0.710	0.754	0.809			RR3		0.732	0.854	0.790		
Online complaints	OC1	0.870	0.739	0.788	0.816	0.872	0.694	OC2		0.794	0.831	0.884			OC3		0.724	0.770	0.792		
Management response	MR1	0.865	0.731	0.846	0.808	0.867	0.686	MR2		0.709	0.759	0.800			MR3		0.793	0.768	0.899		
Perceived risk	PR1	0.860	0.724	0.771	0.864	0.863	0.677	PR2		0.707	0.759	0.858			PR3		0.782	0.832	0.909		
Governance participation	GP1	0.881	0.753	0.794	0.783	0.885	0.719	GP2		0.831	0.897	0.892			GP3		0.729	0.760	0.734		

Tables 1, 2 show the scale properties, e.g., Cronbach’s α value, CITC, Communality, Factor loading and so on for restaurants and consumers, respectively, based on different control elements.

**Table 2:** Scale properties for consumers.

Dimension	Item	Cronbach’s α	CITC	Communality	Factor loading	Composite reliability	AVE
Government regulation	GR1	0.879	0.743	0.776	0.809	0.882	0.715	GR2		0.817	0.760	0.887			GR3		0.748	0.874	0.789		
Restaurant reputation	RR1	0.896	0.821	0.781	0.888	0.898	0.745	RR2		0.783	0.867	0.829			RR3		0.789	0.787	0.809		
Online complaints	OC1	0.876	0.742	0.781	0.818	0.881	0.713	OC2		0.729	0.868	0.800			OC3		0.826	0.779	0.890		
Management response	MR1	0.883	0.750	0.866	0.819	0.886	0.722	MR2		0.817	0.816	0.880			MR3		0.762	0.809	0.800		
Perceived risk	PR1	0.885	0.763	0.789	0.846	0.889	0.727	PR2		0.767	0.803	0.838			PR3		0.814	0.861	0.910		
Governance participation	GP1	0.831	0.665	0.639	0.679	0.834	0.626	GP2		0.740	0.807	0.860			GP3		0.669	0.661	0.702		

The KMO value, communality,^9^ variance interpretation rate value,^10^ and factor loading coefficient value were selected to test the structural validity of the scales, and the KMO values of sample data of restaurants and consumers are 0.850 and 0.878, respectively. In the two groups of sample data, the communality values of all items are higher than 0.4, and the cumulative variance interpretation rates of six dimensions in two scales are 79.789 and 79.573%, respectively. This meets the criterion of a score higher than 70%, indicating that the information content of each dimension can be effectively extracted. The loading values obtained through component matrix rotation for all dimensions in the scales satisfy the criterion of being more than 0.5, indicating that all dimensions can be analyzed as important variables. In addition, the results obtained through component matrix rotation coincide with the scales and dimensions designed in the research plan, indicating that the questionnaire samples have high validity. The above data are shown in Tables 1, 2.

#### 4.3.2. Model fitting degree test

**Figure 3:**
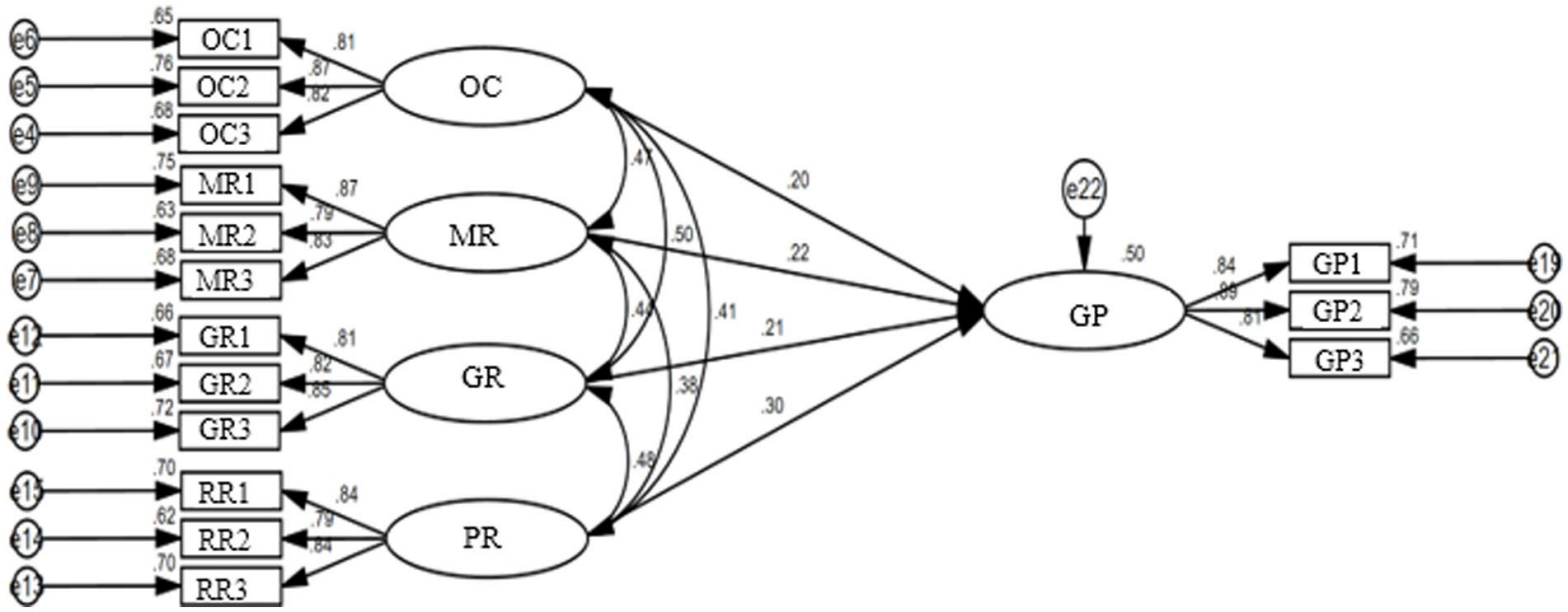
SEM of governance participation willingness of restaurants. OC, Online complaints; MR, Management response; GR, Government regulation; PR, Perceived risk; GP, Governance participation.

**Figure 4:**
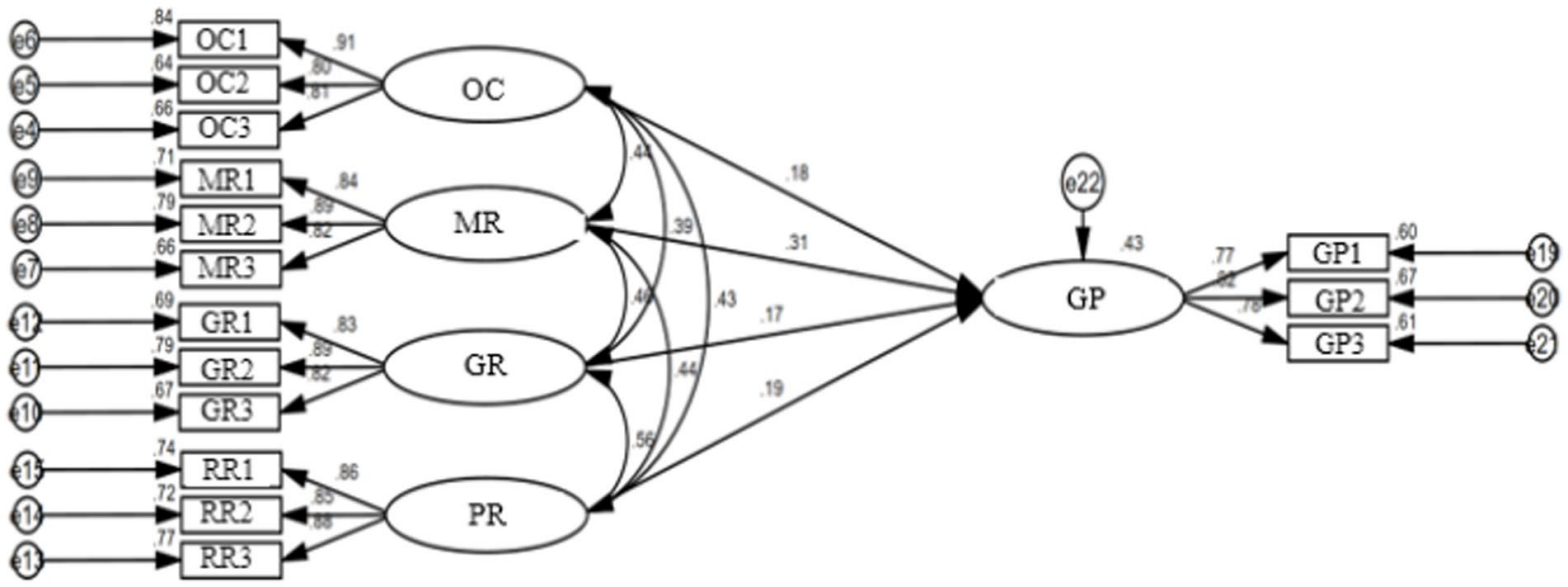
SEM of governance participation willingness of consumers.

The Chi-squared/degrees of freedom (CMIN/DF), normed fit index (NFI), incremental fit index (IFI), Tucker-Lewis index (TLI), comparative fit index (CFI), goodness of fit index (GFI), and root mean square error of approximation (RMSEA) were used to test the fitting degrees of the structural models as shown in Figures 3, 4, and the results are shown in Table 3. Table 3 indicates that the overall fitting degree of the model is satisfying (Gefen et al., 2000), verifying the rationality of the assumed model’s structure.

**Table 3:** Evaluation criteria for overall fitting degree of SEM and fitting evaluation results.

Fit index	CMIN/DF	NFI	IFI	TLI	CFI	GFI	RMSEA
Recommended range	<3	>0.8	>0.9	>0.8	>0.9	>0.8	<0.08
Measured value (Restaurants)	2.427	0.938	0.962	0.950	0.962	0.933	0.065
Measured value (Consumers)	2.919	0.960	0.973	0.964	0.973	0.954	0.056

The table shows the overall fitting degree of the model, in which illustrated by CMIN/DF, NFI and so on.

#### 4.3.3. Test of convergent validity and discriminant validity

According to the assumed SEM, the composite reliability (CR)^11^ and average variance extracted (AVE)^12^ were used as evaluation criteria for convergent validity, and the results are shown in Tables 1, 2. The data show that the CR values of the questionnaires for both restaurants and consumers conform to the criterion of exceeding 0.7, and the AVE values conform to the criterion of exceeding 0.5 (Fornell and Larcker, 1981), indicating that these two questionnaires have good convergent validity.

Discriminant validity refers to a situation when there are multiple different dimensions in a scale, and there are varied differences in observed values between various dimensions so that any two dimensions can be distinguished from each other. In Tables 4, 5, the diagonal lines indicate the square root values of AVEs for various dimensions, and the values below the square root values of AVE are correlation coefficients of this dimension with other dimensions. The results demonstrate that the correlation coefficients between any two of these five dimensions, i.e., government regulation, restaurant reputation, online complaint, management response, and governance participation willingness, are smaller than corresponding square root values of AVE, indicating that both scales for governance participation willingness of restaurants and consumers have good discriminant validity (Fornell and Larcker, 1981).

**Table 4:** Factor correlation coefficient and square root of AVE: restaurants.

Dimension	GR	RR	OC	MR	PR	GP
GR	0.827					
RR	0.480	0.822				
OC	0.498	0.406	0.833			
MR	0.437	0.379	0.475	0.828		
PR	0.191	0.149	0.161	0.142	0.823	
GP	0.546	0.560	0.527	0.516	0.161	0.848

Tables 4, 5 show the square root values of AVEs for various dimensions and also the correlation coefficients of the dimension with other dimensions. These two tables aim to illustrate the discriminant validity of the model.

**Table 5:** Factor correlation coefficient and square root of AVE: Consumers.

Dimension	GR	RR	OC	MR	PR	GP
GR	0.845					
RR	0.558	0.863				
OC	0.390	0.431	0.844			
MR	0.461	0.442	0.439	0.850		
PR	0.300	0.275	0.401	0.335	0.852	
GP	0.485	0.495	0.465	0.547	0.359	0.791

## 5. Results analysis

### 5.1. Statistical characteristics of samples

In this paper, 946 valid questionnaire survey samples were collected, including 337 from restaurant legal persons (operators) and 609 from consumers. Tables 6, 7 give the statistical information of restaurant operator samples and that of consumer samples, respectively. For the restaurant operator questionnaire survey samples, 56.38% of operators were male, 79.23% of operators were aged below 46, and 63.20% of operators have operating time of fewer than 3 years, reflecting the fact that the online restaurant industry is a new format of food consumption only emerging in recent years.

**Table 6:** Individual characteristic information of respondents – restaurant legal persons (operators).

Group	Sample size (*n* = 337)	Proportion (%)
Gender
Male	190	56.38
Female	147	43.62
Age (year)		
18–30	114	33.83
31–45	153	45.40
> 45	70	20.77
Education
Junior high school or lower	63	18.69
High school	78	23.15
Junior college	84	24.93
Bachelor’s degree	104	30.86
Master’s degree or higher	8	2.37
How long have you joined OFD platform such as Meituan or Ele. me to sell online food
Less than 1 year	76	22.55
1–3 years	137	40.65
>3 years	124	36.80

Tables 6, 7 show the statistical characteristic of samples based on restaurants and consumers, respectively. They illustrate general statistical information of the sample.

**Table 7:** Individual characteristic information of respondents – consumers.

Group	Sample size (*n* = 609)	Proportion (%)
Gender
Male	290	47.62
Female	319	52.38
Age (year)		
18–30	246	42.03
31–45	261	42.86
>45	92	15.11
Education
Junior high school or lower	40	6.57
High school	93	15.27
Junior college	102	16.75
Bachelor’s degree	216	35.47
Master’s degree or higher	158	25.94
Occupation
Company employee	206	33.83
Public institution employee	117	19.21
Civil servant	26	4.27
Farmer	34	5.58
Student/graduate student	119	19.54
Self-employed/unemployed/retired	107	17.57
Frequency of Purchasing OFD (per week)
1 time	116	19.05
2 times	150	24.63
3 times	114	18.72
4 times or more	77	12.64
never	152	24.96

For the consumer questionnaire survey samples, female consumers accounted for 52.38%. Over 84% of consumers were aged below 46, and in terms of occupations, most were employees of enterprises and public institutions or students. This coincides with the present situation that OFD is a novel restaurant supply mode with younger demographics being the main consumers (Bates et al., 2020; Roy Morgan Research, 2020; Statista, 2023).

### 5.2. Path analysis of SEM

To test the research hypotheses, path analysis of SEM was performed using the software AMOS. According to the results in Table 8, based on the restaurant samples, it can be seen that government regulation, online complaints, restaurant reputation, and restaurant management responses have significant positive influences on restaurants’ governance participation willingness (*β* = 0.209, *p* < 0.05; *β* = 0.198, *p* < 0.01; *β* = 0.297, *p* < 0.001; *β* = 0.218, *p* < 0.001). Based on the consumer samples, government regulation, online complaints, restaurant reputation, and restaurant management responses similarly have significant positive influences on consumers’ governance participation willingness (*β* = 0.167, *p* < 0.001; *β* = 0.184, *p* < 0.01; *β* = 0.185, *p* < 0.001; *β* = 0.306, *p* < 0.001). Therefore, H_1_, H_2_, H_3_, and H_4_ are upheld.

**Table 8:** Normalized path coefficient of SEM.

Restaurants			Consumers	
Hypotheses	Path	Standardized path coefficient	Path	Standardized path coefficient
$H_{1}$	GR→GP	0.209*	GR→GP	0.167***
$H_{2}$	RR→GP	0.297***	RR→GP	0.185***
$H_{3}$	OC→GP	0.198**	OC→GP	0.184**
$H_{4}$	MR→GP	0.218***	MR→GP	0.306***

The table shows the path coefficient of SEM in accordance with hypotheses in the research. *Sig. at *p* < 0.05; **Sig. at *p* < 0.01; ***Sig. at *p* < 0.001.

### 5.3. Analysis of moderating effects test

The moderating effects of perceived risks were tested using the LMS model by referencing the methods of Kelava et al. (2011) and Jiang et al. (2021). The interactive relationships of four control elements with the perceived risk element were constructed separately; in other words, the respective product terms of government regulation, restaurant reputation, online complaints, and restaurant management responses with perceived risk were obtained. The influences of the product terms on the platform governance participation willingness were observed in order to obtain the results of moderating effects test.

Based on the restaurant samples, Table 9 indicates that the OFD food safety risks perceived by restaurants significantly positively moderate the positive influences of the government regulation, restaurant reputation, online complaints, and restaurant management response on their governance participation willingness (*β* = 0.264, *p* < 0.001; *β* = 0.364, *p* < 0.001; *β* = 0.325, *p* < 0.001; *β* = 0.292, *p* < 0.01); therefore, H_5a_, H_5b_, H_5c_, and H_5d_ are all upheld. Based on the consumer samples, the risks perceived by consumers significantly positively moderate the positive influences of the government regulation and online complaints on consumers’ governance participation willingness (*β* = 0.076, *p* < 0.05; *β* = 0.100, *p* < 0.01), but the influences of the restaurant reputation and restaurant management responses on consumers’ governance participation willingness are not moderated by the perceived risks (*β* = 0.066, *p* > 0.05; *β* = 0.051, *p* > 0.05); therefore, H_5b_ and H_5d_ are not upheld.

**Table 9:** Results of moderating effects test.

Restaurants			Consumers	
Hypotheses	Path	Standardized path coefficient	Path	Standardized path coefficient
$H_{5a}$	GR × PR→GP	0.264***	GR × PR→GP	0.076*
$H_{5b}$	RR × PR→GP	0.364***	RR × PR→GP	0.066
$H_{5c}$	OC × PR→GP	0.325***	OC × PR→GP	0.100**
$H_{5d}$	MR × PR→GP	0.292**	MR × PR→GP	0.051

The table shows the moderating effects of perceived risks of restaurants and consumers in accordance with hypotheses in the research. * Sig. at *p* < 0.05; ** Sig. at *p* < 0.01; *** Sig. at *p* < 0.001.

## 6. Discussion

**Figure 5:**
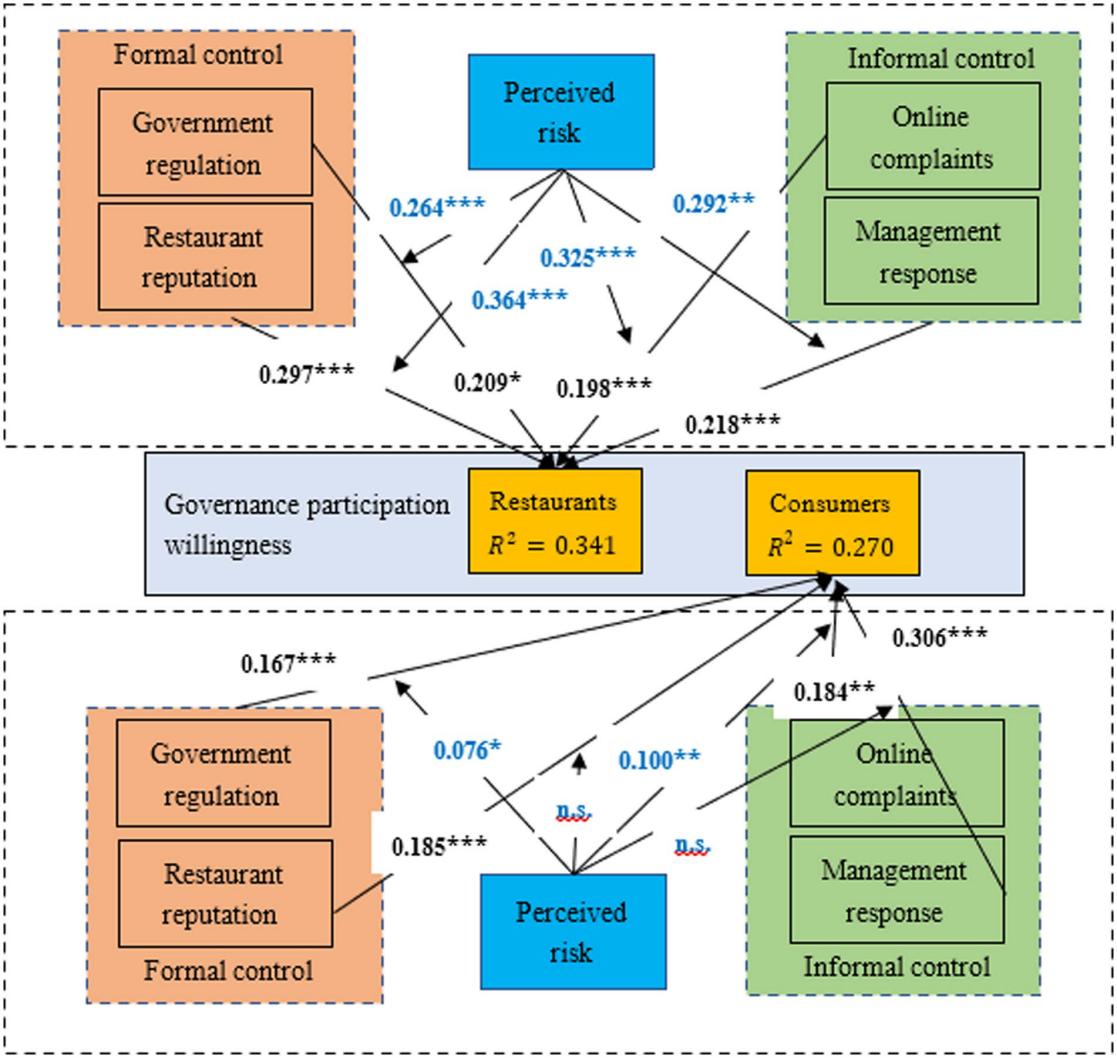
Path analysis results of research model. The figure shows all the path analysis results in accordance with hypotheses in the research. * Sig. at *p* < 0.05; ** Sig. at *p* < 0.01; *** Sig. at *p* < 0.001.

Based on the control theory, this paper has constructed a research framework for the governance participation willingness of restaurants and consumers under the moderating effects of perceived risks. It explores the influences of control elements on the governance participation willingness of both restaurants and consumers and verifies the moderating effects of perceived risk element on the influences. Figure 5 shows the path analysis results of research model, and Table 10 summarizes all hypotheses and corresponding test results.

**Table 10:** Research hypotheses and corresponding results.

Hypotheses	Results	
	Restaurants	Consumers
$H_{1}$ : Government regulation has a positive influence on the governance participation willingness of OFD platform restaurants and consumers.	Supported	Supported
$H_{2}$ : Restaurant reputation has a positive influence on the governance participation willingness of OFD platform restaurants and consumers.	Supported	Supported
$H_{3}$ : Online complaints have a positive influence on the governance participation willingness of OFD platform restaurants and consumers.	Supported	Supported
$H_{4}$ : Restaurant management response has a positive influence on the governance participation willingness of OFD platform restaurants and consumers.	Supported	Supported
$H_{5a}$ : The positive influence of government regulation on the governance participation willingness of OFD platform restaurants and consumers is moderated by perceived risks, and the effect is positively correlated with the perceived risks.	Supported	Supported
$H_{5b}$ : The positive influence of restaurant reputation on the governance participation willingness of OFD platform restaurants and consumers is moderated by perceived risks, and the effect is positively correlated with the perceived risks.	Supported	Not Supported
$H_{5c}$ : The positive influence of online complaints on the governance participation willingness of OFD platform restaurants and consumers is moderated by perceived risks, and the effect is positively correlated with the perceived risks.	Supported	Supported
$H_{5d}$ : The positive influence of restaurant management responses on the governance participation willingness of OFD platform restaurants and consumers is moderated by perceived risks, and the effect is positively correlated with the perceived risks.	Supported	Not Supported

The table shows all hypotheses and corresponding test results of restaurants and consumers, respectively, in the research.

Table 10 indicates that the government regulation positively influences the governance participation willingness of restaurants and consumers and the influences are moderated positively by the food safety risks perceived by restaurants and consumers; in other words, H_1_ and H_5a_ are upheld. This result coincides with the viewpoints of Martin et al. (2017), Ganapati and Reddick (2018), and Jiang et al. (2021) and indicates that government authorities have an irreplaceable role in OFD platform governance. The serious information asymmetry and complex stakeholder network in the online restaurant industry need the government to encourage other platform stakeholders to engage in joint governance while also performing its regulation responsibility.

H_2_ is upheld, indicating that restaurant reputation significantly influences the governance participation willingness of both restaurants and consumers, and this result coincides with the research conclusion of Sthapit (2019); in other words, the implicit incentive of reputation drives consumers’ evaluation participation and motivates restaurants to take corresponding remedy measures against negative evaluations to maintain their own reputation, thereby gaining consumer trust and long-term benefits. For the restaurant samples, the path coefficient of restaurant reputation (*β* = 0.297) is higher than those of government regulation, online complaints, and restaurant management response (*β* = 0.209; *β* = 0.198; *β* = 0.218), indicating that the influence of restaurant reputation on restaurant governance participation willingness is the most significant. Meanwhile, the moderating effect of perceived risks on the positive influence of restaurant reputation on governance participation willingness of restaurants is the most evident (*β* = 0.364), indicating that restaurants consider the influence of reputation as most important; in particular, the stronger the risk perceived by a restaurant, the more likely the restaurant will actively participate in OFD food safety risk governance.

H_3_ and H_4_ were upheld, indicating that online complaints and restaurant management response significantly influence the governance participation willingness of both restaurants and consumers. Consumers’ online complaints provide valuable opinions that restaurants can use to increase their own competitiveness. Thus, a complaint mechanism can drive restaurants to become more involved in platform governance, and this conclusion is consistent with the viewpoint of Dolan et al. (2019). The he positive influence of online complaints exerted by consumers’ perceived food safety risk in moderating consumers’ governance participation willingness is the most evident (*β* = 0.100), indicating that the stronger the risk perceived by a consumer, the more likely the consumer will be to pursue a solution through online complaints, thus driving the restaurant and consumers to more actively participate in governance. The active response of restaurant management to negative evaluation reflects the platform’s attitude, enhancing the communication between consumer and restaurant, and can motivate consumers’ willingness for re-participating in governance, thereby forming a virtuous interaction cycle. Figure 5 shows that for the consumer samples, the path coefficient of restaurant management response (*β* = 0.306) is higher than those of government regulation, restaurant reputation, and online complaints (*β* = 0.167; *β* = 0.185; *β* = 0.184), indicating that the restaurant management response element best drives the governance participation willingness of consumers. This result supports the viewpoint of Xie et al. (2017) that management responses are helpful for platform development.

Based on the questionnaire survey carried out among restaurants, H_5a_, H_5b_, H_5c_, and H_5d_ are all upheld, indicating that the risk perceived by restaurants has a significant positive moderating effect on the relationships between control elements and restaurants’ governance willingness. However, based on the questionnaire survey among consumers, only H_5a_ and H_5c_ are upheld, but H_5b_ and H_5d_ are not; in other words, it was found that the risk perceived by consumers does not moderate the relationships of restaurant reputation and restaurant management response with restaurants’ governance participation willingness. This result indirectly indicates that restaurants are direct beneficiaries of platform governance, especially when they take into account the risks perceived by themselves and consumers for the purpose of pursuing long-term benefits. This coincides with the viewpoint of Hartl et al. (2016).

## 7. Conclusion, policy implications and future work

Given the background of serious information asymmetries in the online restaurant industry, this paper constructed a research framework for analyzing the governance participation willingness of restaurants and consumers under the moderating effects of perceived risks. Drawing on the perspective of control theory, this study examines how different control elements influence the governance participation willingness of restaurants and consumers and analyzes the moderating effects of perceived food safety risks on such willingness. Results show that both formal and informal control elements have significant promoting effects on both restaurant and consumer participation in platform food safety risk governance. Perceived risks have partially significant moderating effects on this relationship. When restaurants perceive strong risks, the control elements can more effectively arouse their governance participation willingness; however, when consumers perceive strong risks, only some of the studied control elements can more effectively arouse consumers’ governance participation willingness. Restaurants consider reputation to best arouse their governance participation willingness, whereas consumers believe that the restaurant management response element best arouses their governance participation willingness.

The research results of this paper have some policy value: Although the government and the platform both play indispensable roles in the OFD food safety risk governance, the relevant effects of restaurants and consumers should not be neglected. The government and platform should encourage consumers to engage in active discussions about restaurants’ service or food safety issues using online complaints or through the platform, so as to motivate restaurants to participate in platform governance for the purpose of protecting their reputation. The OFD platform should urge restaurants well-established on the platform to actively respond to and remedy consumers’ negative evaluations in order to maintain the reputation of the platform and restaurants and further motivate consumers to use the platform and engage in governance. Of course, it must be noted that the data used in this study were from China, and the research results should be further verified with data from other countries.

The “Internet +” new economy has given birth to the online restaurant industry – a novel food consumption format, and driven it to be rapidly developed in the world. The OFD food in the western developed countries, just as that in China, is also produced and processed in a standardized manner and characterized by convenience, and relatively low price, etc., and it also brings huge change of food environment. According to the estimation by Statista (2023), the income of the online restaurant industry will reach 354.1 billion US dollars in China in 2023, which accounts for 38.91% of the global income, and it will be 231.3 billion US dollars in USA and 34.7 billion US dollars in India. China owns the biggest OFD food market in the world. Young population provides the main consumers of OFD food, and China has the largest population of young consumers and the largest population of university undergraduate and graduate students, which are incomparable by other countries in the world. It can be foreseen that OFD industry, including restaurants, will be developed faster in China in the future. In this sense, China’s online restaurant industry is characterized by rapid development, numerous users, large scale, and high penetration, so study on the nutrition quality status of China’s OFD food is unique, proactive, and representative.

Of course, this study has some limitations. For example, the study in this paper explores the influences of formal and informal control elements on the governance willingness of restaurants and consumers separately in theory. However, this paper does not take into account the complementary or substitution effect generated when both formal and informal elements occur simultaneously, in other words, the informal control elements may have moderating effects on the formal control elements. This paper proposes a theoretical framework for collaborative participation by multiple subjects. Owing to the complexity of this framework, the complementary or substitute effect is not taken into further consideration. Therefore, future studies can take this influence element into full consideration. Moreover, the respondents in this paper are restaurants and consumers, for which the reason is that the data on these two are easier to obtain than that on governments. Consequently, future studies can explore other control relationships (e.g., platform and government) in the model with data on governments, to further verify the effectiveness of the model in this paper. In addition, the survey data in this paper is from China, so the research results are to be further verified with data from other countries or other online restaurant platforms.

## Data availability statement

The original contributions presented in the study are included in the article/Supplementary material, further inquiries can be directed to the corresponding author.

## Ethics statement

The studies involving human participants were reviewed and approved by the Ethical Approval was received from the Ethics Committee of Jiangnan University. All methods and procedures in this study were confirmed to the ethics guidelines of the Declaration of Helsinki and followed the ethical standards of the relevant guidelines and regulations. Respondents’ informed consent was obtained on the first page of the questionnaire before commencement of data collection. The patients/participants provided their written informed consent to participate in this study.

## Author contributions

XD, KQ, and LW jointly conceptualized the research study, planned the data collection and analysis, and interpreted data. XD and KQ conducted data collection, data analysis, and drafted the initial manuscript. LW provided oversight and contributed to writing the manuscript. All authors have read and approved this manuscript.

## Funding

LW acknowledged support by the National Social Science Fund of China: Research on social co-governance of food safety risks and cross-border cooperative governance mechanism (grant number 20&ZD117).

## Conflict of interest

The authors declare that the research was conducted in the absence of any commercial or financial relationships that could be construed as a potential conflict of interest.

## Publisher’s note

All claims expressed in this article are solely those of the authors and do not necessarily represent those of their affiliated organizations, or those of the publisher, the editors and the reviewers. Any product that may be evaluated in this article, or claim that may be made by its manufacturer, is not guaranteed or endorsed by the publisher.

## Footnotes

^1^"Ghost kitchens," also called "Cloud kitchens" or "Dark kitchens," refers to a restaurant that does not have a dining area and waitstaff but only provides food for distribution (Li et al., 2020).

^2^Opportunistic behavior refers to behaviors whereby, in the case of information asymmetry, people do not completely and/or truthfully disclose all information and are engaged in other activities benefiting themselves at the cost of others, such as false advertisement, patent plagiarism, violation of laws and regulations, and tax evasion (Williamson, 1985).

^3^Platform ecosystem is an economic community centered in a platform of environment or condition that can provide a service to consumers and formed by integrating other enterprises that provide resources needed for consumers in consumption (Tiwana, 2018).

^4^A bilateral market refers to a market in which two groups of stakeholders need to perform transactions through an intermediate layer or platform and the income of one group depends on the quantity of the other group of stakeholders (Armstrong, 2006).

^5^A CITC test can identify the correlation of each variable with the population, and a variable with a CITC value less than 0.4 needs to be deleted.

^6^If the Cronbach’s coefficient is more than 0.7, it indicates that the reliability is high. If the Cronbach’s coefficient is less than 0.35, it indicates that the reliability is low, so the variable should be deleted (Guielford, 1965).

^7^A KMO test will determine the inter-variable correlation by comparing the simple correlation coefficient and partial correlation coefficient between various variables. If the partial correlation coefficient is less than the simple correlation coefficient, the correlation is stronger. This is used to determine the suitability degree of information extraction. The closer the KMO value is to 1, the higher the suitability. It is generally believed that KMO values of over 0.9, 0.8–0.9, 0.6–0.8, 0.5–0.6, and below 0.5 represent very suitable, relatively suitable, general, unsuitable, and extremely unsuitable, respectively (Kaiser, 1974).

^8^The statistical meaning of factor loading is the correlation of the *i*th variable with the *j*th common factor (dimension), that is, the weight that *Xi* depends on *Fj*. Therefore, the factor loading coefficient is used to measure the corresponding relationship between a factor (dimension) and a variable, and the criterion for this value is more than 0.5 (Hair et al., 1998).

^9^Communality, also called "common variance," represents the information content of an item, which can be extracted. The higher the communality, the higher the degree of interpretation of the indicator by principal component, and the more the information content extracted. The criterion is generally taken as 0.4.

^10^Variance interpretation rate is used to show how much original data a factor (dimension) contains, that is, the information extraction level. A cumulative variance interpretation rate usually ranges from 70 to 90% (Jolliffe, 2002).

^11^Composite reliability refers to the reliability of a composite score (a new score composed of the sum of more than one scores) (Fornell and Larcker, 1981).

^12^Average variance extracted is a statistic used to test the internal consistency of a structural variable in statistics (Fornell and Larcker, 1981).
