# Supplementary material for: Study on effect of collaborative governance participation willingness of online food delivery platform restaurants and consumers from perspective of control theory: Based on moderating effects of perceived risks
Source: Front Psychol. 2023 Mar 14;14:1149538. doi: 10.3389/fpsyg.2023.1149538 (PMC10043407; doi:10.3389/fpsyg.2023.1149538)
Supplement: Supplementary file 1 [file Table_1.DOCX]

**Table1: Questionnaire contents- to restaurants**

| **Dimension** | **Symbol** | **Item** |
| --- | --- | --- |
| Government Regulation | GR1 | The government should issue laws and regulations on the governance of OFD platforms, and put forward targeted guidance. Do you agree? |
|  | GR2 | The government should assume the responsibility of supervision and promote the compliance operation of OFD. Do you agree? |
|  | GR3 | The government should assume the responsibility of supervision and try its best to prevent the emergence of food safety and quality problems of OFD. Do you agree? |
| Restaurant reputation | RR1 | Restaurants in Meituan's delivery platform are recognized by the industry. Do you agree? |
|  | RR2 | Restaurants in Meituan's delivery platform have a high reputation in the industry. Do you agree? |
|  | RR3 | Restaurants in Meituan's delivery platform have a good public image. Do you agree? |
| Online complaints | OC1 | When consumers encounter problems with the quality and safety of OFD, they will complain to you (" You "here refers specifically to the restaurants and operators who sell food to consumers through the OFD platform, and the questionnaires are filled out by the representatives of the restaurants and operators, the same below) or the platform. Whether you agree. |
|  | OC2 | Consumers will complain to you or the OFD platform when they encounter problems with the food delivery service of the OFD platform. Whether you agree. |
|  | OC3 | When consumers encounter food safety and quality problems, they will ask you or the OFD platform to solve the problems and give suggestions for improvement. Whether you agree. |
| Management response | MR1 | Restaurants will respond quickly to consumers’ complaints. Whether or not you do that? |
|  | MR2 | Restaurants will explain the "bad reviews" from consumers and reduce the rate of bad reviews. Whether you will do so. |
|  | MR3 | If the customer gives a bad review and responds to the problem, the restaurants will provide improvement plan and give appropriate financial compensation (such as discount or coupon) |
| Perceived risk | PR1 | Eating OFD will not bring food safety problems. Do you agree? |
|  | PR2 | Do you support the view that eating OFD will not bring negative health effects to consumers |
|  | PR3 | Eating OFD will not make consumers lack of daily dietary nutrition. Do you agree? |
| Governance participation | GP1 | According to the consumers, there are some problems in the food safety of OFD, and comprehensive management needs to be strengthened. In the governance process of OFD platform, the participation of multiple subjects (including the platform government, consumers and restaurants) can effectively control the disputes of takeaway catering platform. Do you agree? |
|  | GP2 | In the governance process of OFD platform, the participation of multiple subjects (including the platform government, consumer merchants) will help the OFD platform reduce food safety and quality problems. Do you agree? |
|  | GP3 | In the governance process of OFD platform, the participation of multiple subjects (including platform, government, consumers and merchants) is conducive to the healthy and stable development of the OFD platform. Do you agree? |

**Table 2: Questionnaire contents- to consumers**

| **Dimension** | **Symbol** | **Item** |
| --- | --- | --- |
| Government Regulation | GR1 | The government should issue laws and regulations on the governance of OFD platforms, and put forward targeted guidance. Do you agree? |
|  | GR2 | The government should assume the responsibility of supervision and promote the compliance operation of OFD. Do you agree? |
|  | GR3 | The government should assume the responsibility of supervision and try its best to prevent the emergence of food safety and quality problems of OFD. Do you agree? |
| Restaurant reputation | RR1 | Restaurants in Meituan's delivery platform are recognized by the industry. Do you agree? |
|  | RR2 | Restaurants in Meituan's delivery platform have a high reputation in the industry. Do you agree? |
|  | RR3 | Restaurants in Meituan's delivery platform have a good public image. Do you agree? |
| Online complaints | OC1 | If you encounter food safety and quality problems, will you complain to the restaurants or the delivery platform? |
|  | OC2 | When you encounter delivery service problems, will you complain to the restaurants or the delivery platform? |
|  | OC3 | When encountering food safety and quality problems, will you ask the restaurants or delivery platforms to solve the problems and give improvement suggestions? |
| Management response | MR1 | Based on your observation and experience, do restaurants or delivery platforms respond quickly to user comments? Do you agree? |
|  | MR2 | According to your observation and experience, whether the restaurants or delivery platforms explain the "bad reviews" put forward by consumers to reduce the bad review rate. Do you agree? |
|  | MR3 | According to your observation and experience, will the restaurants or delivery platforms provide improvement plans and appropriate financial compensation (such as discounts or coupons) for the problems reported by consumers given "bad reviews"? Do you agree? |
| Perceived risk | PR1 | Eating OFD will not bring food safety problems. Do you agree? |
|  | PR2 | Do you support the view that eating OFD will not bring negative health effects to consumers |
|  | PR3 | Eating OFD will not make consumers lack of daily dietary nutrition. Do you agree? |
| Governance participation | GP1 | There are some problems in the food safety of OFD, which need to strengthen comprehensive treatment. In the governance process of OFD platforms, the participation of multiple subjects (including platforms, governments, consumers and merchants) can effectively control disputes on OFD platforms. Do you agree? |
|  | GP2 | In the governance process of OFD platform, the participation of multiple subjects (including the platform government, consumer merchants) will help the OFD platform reduce food safety and quality problems. Do you agree? |
|  | GP3 | In the governance process of OFD platform, the participation of multiple subjects (including platform, government, consumers and merchants) is conducive to the healthy and stable development of the OFD platform. Do you agree? |

**Table 3: Results of CR of pre-survey**

| **Item** | **CR: Restaurants** | **CR: Consumers** |
| --- | --- | --- |
| GR1 | 9.286*** | 8.645*** |
| GR2 | 9.989*** | 13.531*** |
| GR3 | 10.530*** | 6.894*** |
| RR1 | 12.478*** | 8.239*** |
| RR2 | 5.785*** | 13.678*** |
| RR3 | 6.574*** | 4.582*** |
| OC1 | 4.890*** | 9.463*** |
| OC2 | 4.509*** | 4.216*** |
| OC3 | 9.758*** | 11.467*** |
| MR1 | 12.163*** | 13.096*** |
| MR2 | 8.069*** | 11.785*** |
| MR3 | 5.897*** | 11.090*** |
| PR1 | 11.368*** | 6.354*** |
| PR2 | 13.096*** | 3.309*** |
| PR3 | 4.859*** | 8.930*** |
| GP1 | 5.989*** | 4.205*** |
| GP2 | 15.749*** | 11.567*** |
| GP3 | 9.000*** | 5.989*** |

**Table 4: Results of CITC and Cronbach’s α of pre-survey**

| **Dimension** | **Item** | **CITC** | | **Cronbach’s α after items deleted** | | **Cronbach’s α** | |
| --- | --- | --- | --- | --- | --- | --- | --- |
|  |  | **Restaurants** | **Consumers** | **Restaurants** | **Consumers** | **Restaurants** | **Consumers** |
| Government Regulation | GR1 | 0.642 | 0.787 | 0.827 | 0.842 | 0.873 | 0.883 |
|  | GR2 | 0.773 | 0.678 | 0.776 | 0.853 |  | |
|  | GR3 | 0.564 | 0.679 | 0.841 | 0.766 |  | |
| Restaurant reputation | RR1 | 0.778 | 0.702 | 0.821 | 0.756 | 0.850 | 0.801 |
|  | RR2 | 0.774 | 0.765 | 0.841 | 0.797 |  | |
|  | RR3 | 0.807 | 0.765 | 0.763 | 0.744 |  | |
| Online complaints | OC1 | 0.787 | 0.698 | 0.806 | 0.751 | 0.869 | 0.790 |
|  | OC2 | 0.765 | 0.790 | 0.801 | 0.784 |  | |
|  | OC3 | 0.754 | 0.777 | 0.826 | 0.745 |  | |
| Management response | MR1 | 0.873 | 0.802 | 0.771 | 0.732 | 0.834 | 0.792 |
|  | MR2 | 0.818 | 0.831 | 0.831 | 0.763 |  | |
|  | MR3 | 0.854 | 0.845 | 0.811 | 0.756 |  | |
| Perceived risk | PR1 | 0.546 | 0.798 | 0.814 | 0.750 | 0.887 | 0.799 |
|  | PR2 | 0.576 | 0.781 | 0.829 | 0.744 |  | |
|  | PR3 | 0.604 | 0.674 | 0.760 | 0.709 |  | |
| Governance participation | GP1 | 0.798 | 0.698 | 0.746 | 0.791 | 0.799 | 0.830 |
|  | GP2 | 0.790 | 0.804 | 0.773 | 0.719 |  | |
|  | GP3 | 0.734 | 0.712 | 0.765 | 0.789 |  | |
